# Retreatment with Brentuximab Vedotin in Patients with Relapsed/Refractory CD30+ Malignancies: A Retrospective Medical Chart Review Study in Spain-The BELIEVE Study

**DOI:** 10.3390/cancers17071137

**Published:** 2025-03-28

**Authors:** Anna Sureda, Ramón García-Sanz, Eva Domingo-Domenech, Francisco Javier Capote, Antonio Gutiérrez, Antonia Rodriguez, David Aguiar, Pilar Giraldo, María Stefanía Infante, Javier López-Jiménez, Carmen Martínez, Blanca Sánchez-González, Pablo L. Ortiz-Romero, Marta Grande, Lourdes Baeza-Montañez

**Affiliations:** 1 Intitut Català d’Oncologia, Duran i Reynals Hospital, IDIBELL, 08908 Hospitalet de Llobregat, Spain; asureda@iconcologia.net (A.S.); edomingo@iconcologia.net (E.D.-D.); 2 CIBERONC, Institute of Cancer and Hematological Diseases, Gregorio Marañón General University Hospital, FiBGM, Dr. Esquerdo 46, 28007 Madrid, Spain; 3 Puerta del Mar University Hospital, 11009 Cádiz, Spain; fjcapote@hotmail.es; 4 Son Espases University Hospital, IdISBa, 07120 Islas Baleares, Palma, Spain; antoniom.gutierrez@ssib.es; 5 Hematology Department, 12 De Octubre University Hospital, 28041 Madrid, Spain; antonia.rodriguez@salud.madrid.org; 6 Gran Canaria University Hospital Dr. Negrin, 35010 Las Palmas de Gran Canaria, Spain; dagubuj@gobiernodecanarias.org; 7 Quironsalud Zaragoza Day Hospital, 50012 Zaragoza, Spain; giraldocastellano@gmail.com; 8 Infanta Leonor University Hospital, 28031 Madrid, Spain; ms.infante@gmail.com; 9 Ramón y Cajal University Hospital, 28034 Madrid, Spain; jlopezj.hrc@salud.madrid.org; 10 Hospital Clínic de Barcelona, 08036 Barcelona, Spain; cmarti@clinic.cat; 11 Del Mar University Hospital, 08003 Barcelona, Spain; bsanchezgonzalez@parcdesalutmar.cat; 12 Dermatology Department, 12 De Octubre University Hospital, Institute i+12, Medical School, Complutense University, 28040 Madrid, Spain; pablo.ortiz@salud.madrid.org; 13 Medical Departament, Takeda Farmacéutica España S.A., 28027 Madrid, Spain; marta.grande@takeda.com (M.G.); lourdes.baeza@takeda.com (L.B.-M.); 14 Clinical Medicine, Alcalá University, 28801 Alcalá de Henares, Spain

**Keywords:** brentuximab vedotin, RWE, retreatment, Hodgkin lymphoma, systemic anaplastic large cell lymphoma, cutaneous T cell lymphoma

## Abstract

Background/Objectives: Brentuximab vedotin (BV) has been shown to be effective in the treatment of classical Hodgkin’s lymphoma (cHL), systemic anaplastic large cell lymphoma (sALCL), and CD30-positive cutaneous T-cell lymphoma (CTCL). This study aimed to evaluate the effectiveness and safety of BV retreatment in patients with relapsed/refractory cHL, sALCL, and CD30-positive CTCL. Methods: This multicenter, non-interventional, retrospective medical chart review study analyzed medical records from 43 patients retreated with BV in Spain. Patients were included if they had relapsed or refractory cHL, sALCL, or CD30-positive CTCL and were previously treated with BV. Demographic characteristics, disease stage, response to treatment, survival outcomes, and adverse events were analyzed. Results: The study population included 16 patients with cHL, 13 with sALCL, and 14 with CTCL. The majority were male (58.1%) with a mean age of 46.2 years and baseline ECOG scores of 0–1. Among cHL and sALCL patients, disease stage, according to the Ann Arbor classification, ranged from I to IVB, while in CTCL, EORTC clinical stages ranged from IA to IVB. The overall response rate to BV retreatment was 76.7%, with the highest response observed in sALCL (92.3%). Complete remission was achieved in 60.5% of patients. Median progression-free survival was 25.4 months, and overall survival reached 50 months. Treatment failure occurred in 37.2% of patients. BV was generally well tolerated, with peripheral neuropathy being the most frequently reported adverse event. Conclusions: The BELIEVE study is the largest study to date demonstrating that retreatment with BV is an effective and well-tolerated option for patients with relapsed or refractory CD30-positive malignancies.

## Simple Summary

Patients with certain types of blood cancers that express the CD30 protein, such as classical Hodgkin’s lymphoma, systemic anaplastic large cell lymphoma, and cutaneous T-cell lymphoma, often experience relapses or resistance to treatment. Brentuximab vedotin is a targeted therapy that has shown effectiveness in these cancers. This study, conducted in Spain, evaluated the effectiveness and safety of retreatment with brentuximab vedotin in 43 patients who had relapsed or refractory disease. The results showed that 76.7% of patients responded to the therapy, with the highest response rate in those with systemic anaplastic large cell lymphoma. More than half of the patients achieved complete remission. The treatment was generally well tolerated, with peripheral neuropathy being the most common side effect. These findings suggest that retreatment with brentuximab vedotin can be a valuable option for patients who need additional therapy after relapses or displayed resistance to previous treatments.

## 1. Introduction

Brentuximab vedotin (BV) is an antibody–drug conjugate that targets CD30. It comprises a humanized IgG monoclonal antibody linked to the antimitotic agent monomethyl auristatin E (MMAE), via a cleavable valine–citrulline linker. Upon binding to CD30, BV is internalized and the MMAE component is released, disrupting the microtubule network and subsequent apoptotic cell death of CD30-expressing tumor cells [1]. BV has demonstrated significant efficacy in treating relapse/refractory CD30-positive malignancies, particularly classical Hodgkin lymphoma (cHL) and the NHL subtypes [2], systemic anaplastic large cell lymphoma (sALCL), and cutaneous T-cell lymphoma (CTCL) [3,4,5,6,7].

HL accounts for approximately 15% of all lymphomas [8], with cHL, typically CD30+, being the most common subtype (95% of cases), peaking in early adulthood and later in life (>55-year-old adults) [8]. Advances in chemotherapeutic regimens, including anti-CD30 therapy, anti-PD1 therapy, and radiotherapy, have significantly improved the prognosis of HL, achieving high cure rates [9]. However, relapsed/refractory disease is still a problem that is difficult to manage, particularly in the late stages of the disease [8,9].

sALCL comprises approximately 2–8% of all non-Hodgkin lymphomas [10]. The population involved vary depending on the expression of ALK, with children and young adults mainly affected by ALK-positive sALCL, and older populations affected by ALK-negative sALCL [2]. Standard chemotherapy provides a poor prognosis, with recurrence rates of the disease as high as 40–65%, although the addition of BV along with chemotherapy in first-line settings improves the outcome [11].

CTCLs represent only 4% of all NHL cases, with mycosis fungoides (MF) as the most common type, followed by Sézary syndrome and CD30-positive primary cutaneous ALCL (pcALCL) [2,12]. These lymphomas mainly affect adults, with a median age at diagnosis between 50 and 70 years and a higher incidence in males [12]. Patients with CTCL often develop relapsed or refractory disease, and while systemic therapy can offer active responses, these are often short-lived [12].

All these types of lymphomas can be treated with BV at different moments of their evolution. If disease progression occurs after stopping therapy, resuming BV treatment could be considered [1]. However, few published studies have evaluated the efficacy and safety of BV as retreatment in cHL and sALCL, and only few clinical cases in CTCL have been studied [3,4,5,6,7,13,14]. Results from these studies have demonstrated that cHL, sALCL and CTCL patients can achieve good responses from BV retreatment, with an overall response rate (ORR) above 50% [3,4,5,6,7]. Altogether, these studies have provided no sufficient data regarding the effectiveness and safety of BV retreatment. This study aims to address this gap by collecting data from real-world outcomes and management in routine clinical practice in Spain by evaluating the effectiveness and safety of BV retreatment in a cohort of Spanish patients with relapsed/refractory CD30-positive lymphomas.

## 2. Materials and Methods

### 2.1. Study Design and Population

**Figure 1:**
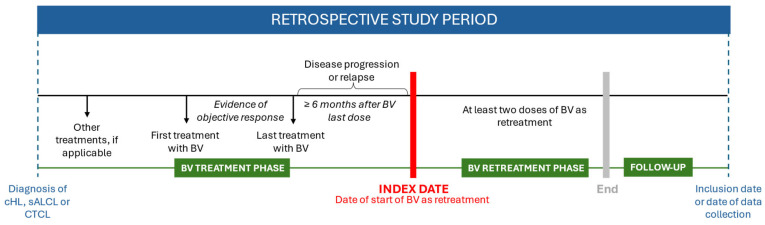
Study flow chart and periods. BV: brentuximab vedotin; cHL: classical Hodgkin lymphoma; CTCL: cutaneous T-cell lymphoma; sALCL: systemic anaplastic large cell lymphoma.

This multicenter, non-interventional, retrospective medical chart review study was conducted across 30 public and private centers in Spain. The study focused on adult patients histologically confirmed to have cHL, sALCL, or CD30-positive CTCL (including MF and pcALCL). These patients had previously received a BV-containing regimen, achieved complete (CR) or partial remission (PR), and subsequently experienced disease relapse or progression occurring at least six months after the last dose of the initial BV treatment. Additionally, these patients experienced disease progression or relapse after discontinuing last treatment. Patients must have received at least two doses of BV during the retreatment period, with at least six months of follow-up data or until discontinuation due to toxicity or death. A diagram of study periods is shown in Figure 1.

### 2.2. Data Sources and Measurements

Variables were analyzed retrospectively and based on the information available in the medical records for the selected patients since their diagnoses, following standard clinical practices. The variables analyzed included: demographic information at diagnosis, disease characteristics before BV-retreatment, treatment history and characteristics, response to BV during the retreatment, follow-up outcomes, and safety outcomes.

The primary objective was to assess the ORR following BV retreatment, defined as the percentage of patients achieving CR or PR, based on PET-CT findings when applicable. Additionally, safety was evaluated by recording adverse events such as peripheral neuropathy, neutropenia, febrile neutropenia, renal dysfunction, liver dysfunction, pulmonary disorders, and infusion reactions, graded according to the NCI-CTCAE Version 5.0 [15].

Secondary objectives included the analysis of overall survival (OS), best overall response (BOR, including CR, PR, stable disease and disease progression), duration of response (DOR, calculated as the time from first documented CR or PR until progression or death) based on PET-TC status, when applicable, progression-free survival (PFS), and time to treatment failure (TTF), defined as the time from the index date to the discontinuation of treatment due to inadequate response to therapy, intolerable side effects or toxicity, disease progression, and patient withdrawal for whatever reason.

### 2.3. Statistics

Statistical analyses were conducted by IQVIA Information S.A. using SAS^®^ software version 9.4. This was largely a descriptive study, with few analyses. Descriptive statistics for discrete variables were reported as absolute and relative frequencies, while quantitative variables were presented with mean, standard deviation, median, quartiles, and range (minimum and maximum). Normality of data distribution was evaluated using the Shapiro–Wilk test. Paired t-tests were applied to compare follow-up and baseline values for normally distributed data, while the Wilcoxon signed-rank test was employed for non-normally distributed data. Categorical variables were analyzed using Fisher’s exact test. Correlation analyses were performed using the Pearson or Spearman coefficients, depending on the linearity of the relationship, with simple linear regression also being utilized. A two-sided 95% CI (α = 5%) was considered by default unless otherwise specified in the description of the analyses.

Missing values and drop-outs were included in the description of variables, but no imputation was considered for this study.

### 2.4. Treatment

The administration of BV was performed in accordance with the specifications outlined in the Summary of Product Characteristics (SmPC) [1]. The mean initial dose for the first BV treatment as well as for BV retreatment was 1.8 mg/kg. This dose was adjusted based on the clinician criteria and due to adverse events when applicable. During the retreatment period, all patients received at least two doses of BV after ≥6 months of the last dose of BV in the treatment period.

## 3. Results

### 3.1. Patient Characteristics

A total of 51 patients were eligible for the analysis set, but 8 of them were excluded because they did not meet the inclusion criteria. The remaining 43 patients were included in the study, 16 with cHL, 14 with CTCL, and 13 with sALCL. CTCL patients included 12 MFs and only 2 pcALCLs, so they were aggregated as CTCLs throughout the study. Demographic and baseline characteristics are summarized in Table 1. Overall, 58.1% of patients were male (56.2% in cHL, 57.1% in CTCL, and 61.5% in the sALCL group, respectively), with a mean age (SD) at first visit of 46.2 (14.6) years. The majority (88.1%) were Caucasian, and most presented an ECOG performance status of 0–1 prior to BV retreatment.

Prior to BV retreatment, in the context of cHL and sALCL and according to the Ann Arbor classification, four patients were classified as stage I, eight as stage II, four as stage III, and nine as stage IVA and B. For the CTCL group, the EORTC clinical stages of the patients were as follows: two patients in stage IA or IB, three in stage IIB, two in stage IVA, and four in stage IVB.

All patients were CD30-positive at diagnosis.

### 3.2. Treatment Patterns

The median (SD) initial dose of first BV treatment and BV retreatment was 1.8 (0.2) mg/kg. The mean (SD) number of treatments received by patients before BV retreatment was 6.2 (4.8) (Table 2). Six (15%) patients had an initial dose adjustment (median 1.2 (0.1) mg/kg) mainly due to peripheral neuropathy. The median time from the last BV treatment to relapse was 19.4 months, with a shorter median time observed in CTCL patients (11.2 months) compared to cHL (12.9 months) and sALCL (17.2 months).

After BV retreatment, the mean (SD) number of treatments received by patients was 1.1 (1.8), with 8 (19%) patients needing an initial dose adjustment mostly due to peripheral neuropathy, including 1 sALCL patient due to paresthesia. Another dose adjustment was performed in one cHL patient due to cytopenia. The median (SD) of new dose adjustments was 1.2 (0.1) mg/kg, although 1 sALCL patient needed a dose adjustment to 1.0 mg/kg.

Regarding transplants, 20.9% of patients had received an autologous stem cell transplantation (SCT) before starting the first treatment with BV, and 23.3% patients underwent autologous SCT between the first BV treatment and BV retreatment. Six patients (14%) had undergone allogenic SCT before BV retreatment, and 20.9% of patients underwent allogenic SCT after retreatment (Table 3).

### 3.3. Overall Response Rate (ORR)

**Figure 2:**
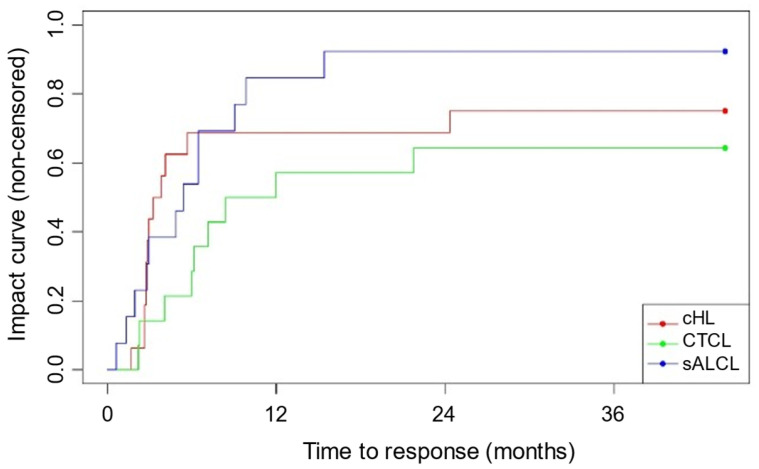
Kaplan–Meier estimates of ORR.

The ORR following BV retreatment was achieved by 33 patients (76.7%), with a median time to achieve response of 4.1 months (IQR, 2.8–6.5). Substantial variability between the different lymphoma subtypes was observed. Patients with sALCL exhibited the highest ORR (92.3%), followed by cHL patients (75%) and CTCL patients (64.3%). The median time to response was 2.9 (IQR, 2.7–4.0), 6.2 (4.1–8.4), and 5.1 (2.4–7.8) months for cHL, CTCL and sALCL groups, respectively (Figure 2).

### 3.4. Best Overall Response (BOR)

**Figure 3:**
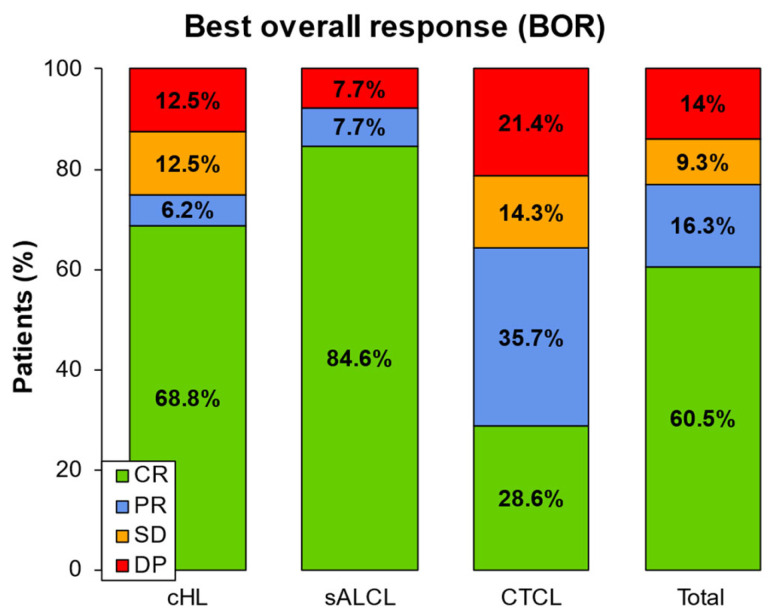
BOR after BV retreatment. N: 43 patients (16 cHL, 13 sALCL, and 14 CTCL). BV: Brentuximab vedotin; CR: complete response; DP: disease progression; PR: partial response; SD: stable disease.

Regarding BOR, 60.5% of patients achieved CR, with the following percentages: 84.6% in the sALCL group, 68.8% in cHL, and 28.6% in CTCL. PR was achieved by seven patients (16.3%): one cHL, one sALCL and 5 CTCL. Finally, 4 patients (9.3%) experienced stable disease, while 14% (6/43) progressed (2 cHL, 1 sALCL and 3 CTCL) (Figure 3). Overall, the median (IQR) time to achieve CR in 26 patients was 4 (2.8–7.2) months, being 3 (2.8–4.1) months in cHL patients, 5.4 (2.8–9.1) months in the sALCL group, and 7.8 (4.7–15.1) months in CTCL patients (Figure S1). The duration of response (DOR) varied, with cHL patients maintaining their response for a median of 39.4 (IQR, 19.3–59.5) months (*n* = 4), 8.9 months for sALCL (7.8–9.5) (*n* = 5), and 3.8 months (0.8–14.6) for CTCL (*n* = 4).

### 3.5. Progression Free Survival (PFS), Time to Treatment Failure (TTF), and Overall Survival (OS)

**Figure 4:**
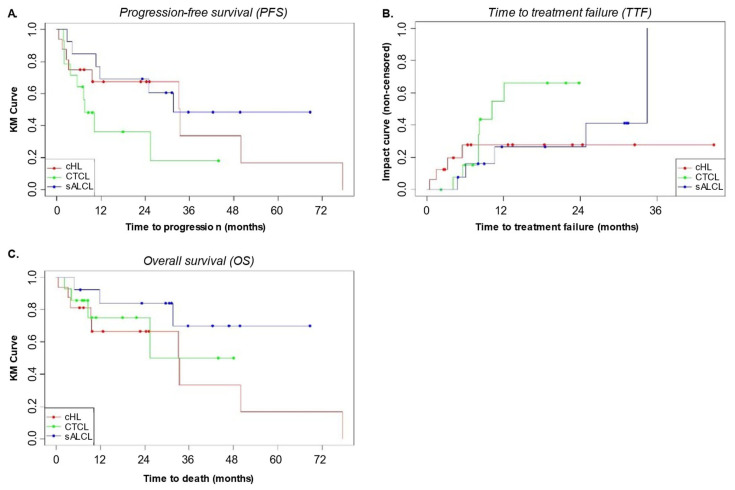
Kaplan–Meier estimates of PFS (**A**), TTF (**B**), and overall survival (**C**) in the overall population. OS: overall survival; PFS: progression free survival; TTF: time to treatment failure.

Overall, the median PFS (IQR) was 25.4 (2.7–25.2) months, being 33.4 (2.6–33.4) months for cHL, 31.6 (4.1–24.9) for sALCL and 7.6 (2.1–7.6) months for CTCL (Figure 4A). After 2 years from the index date, survival without progression was observed in 44.2% of overall patients, being achieved by 43.8% (7/16) of patients with cHL, 35.7% (5/14) of patients with CTCL and 53.8% (7/13) of patients with sALCL.

Treatment failure was observed in 16 patients (37.2%) with a median (IQR) TTF of 7.0 (4.4–10.4) months (Figure 4B).

The median OS (IQR) was 50.0 months (4.0–32.4) (Figure 4C) with 27 patients (62.8%) alive. After 2 years from the index date, the highest percentage of patients who survived was observed in the sALCL patient group (76.9%, 10/13. Median OS not reached (IQR 4.9–31.6)), followed by CTCL patients (71.4%, 10/14. Median OS of 25.4 (3.2–17.0) months) and cHL (43.8%, 7/16. Median OS of 33.1 (3.9–33.4) months) patients.

### 3.6. Safety

BV retreatment was generally well-tolerated, with most adverse events being non-severe. In total, 18 patients (45%) experienced AEs related to BV retreatment: 7 cHL, 7 sALCL, and 4 CTCL. The most common AE was peripheral neuropathy (25.6%; two patients with grade 2, two with grade 3, and one patient with grade 4), followed by other AEs of clinical relevance (23.3%), neutropenia (11.6%, one grade 2 and two grade 3 patients), and pulmonary disorders (2.3%).

Other AEs with clinical relevance (23.3% of overall patients) included an increase in ALT/AST, anemia, bacterial and viral infections, catheter bacteriemia and related-thrombosis, fever, diarrhea, asthenia, hand and neck rash, and muscle contracture.

Overall, severe AEs were reported in eight patients (18.6%): two cHL, three CTCL, and three sALCL patients (Table 4). There were four grade 3 events reported in three patients and one grade 4 event reported. No grade 5 events were reported.

In patients with AEs, the mean (SD) number of cycles during BV retreatment was higher (9.2 (5.7)) compared to patients without AEs (6.3 (3.8)). This was replicated in the analysis by patient subgroups, with the mean number of BV retreatment cycles being higher in cHL, sALCL, and CTCL patients (7.3 (6.0),10.6 (6.6) and 10.2 (2.8), respectively) with AEs compared to patients without AEs (cHL 6.5 (5.8), sALCL 5.7 (2.0), and CTCL 6.6 (3.6)).

## 4. Discussion

The BELIEVE study provides valuable insights into the effectiveness and safety of BV retreatment in a real-world setting. These findings underline BV’s role as a useful therapeutic option for patients with relapsed or refractory CD30-positive malignancies, particularly those with cHL, sALCL, and CTCL.

The ORR of 76.7% observed in this study aligns with previously reported ORRs in clinical trials and smaller studies with BV as retreatment [3,4,5,13,16,17,18,19,20,21,22]. In a phase II pivotal study of BV in patients with relapsed or refractory CD30-positive Hodgkin’s lymphomas, the ORR was 75% with CR in 34% of patients [21], which is replicated in the current study, where the ORR was the same, with a high number of HL patients achieving CR as a best response (68.8%). These results are also reproduced in sALCL patients. The high ORR (92%) and CR (85%) rates in sALCL patients shown in this study are also in line with previous results from a phase II trial (NCT00866047) in which ORR was 86% with 66% of patients achieving CR [23], as well as those from a phase III trial (NCT01777152), in which ORR was 83% with 68% of patients achieving CR [18]. These results seem to suggest that sALCL would benefit the most from BV retreatment, but in-depth analyses are needed to confirm this.

For patients with CTCL, the ORR results of this study are very promising, with an ORR of 64.3% and 28.6% of patients achieving CR. Again, these results are aligned with the phase III ALCANZA trial in CD30-positive CTCL patients (ORR of 54.7%, CR of 17.2%) [19,24] and the data from the Spanish Primary Cutaneous Lymphoma Registry (ORR to retreatment of 54%, CR of 23%) [14], which provides evidence that BV can help to significantly improve the prognosis of these patients.

The complexity of treating this patient cohort is underlined by the extensive number of previous treatment lines (a median of 5), including a significant number of transplants. Nearly 21% of patients had undergone an autologous SCT before the first exposition to BV treatment, with an additional 20.9% of all cases being consolidated with allogeneic transplants after BV retreatment. Again, this highlights a relevant role for BV as a bridging therapy for heavily pretreated patients, towards potentially curative treatments such as allogeneic SCT. Several studies have shown that BV can achieve a significant number of remissions before transplantation, improving their outcomes and long-term survival [17]. For many patients, reaching CR during BV treatment could be the only opportunity to proceed with allogeneic SCT as a curative option [9]. Moreover, the observed median TTF of 7.0 months, PFS of 25.4 months, and OS of 50.0 months further reinforce the potential of BV retreatment for extending survival in these patients. These findings are especially relevant for clinicians, as retreatment options in relapsed/refractory settings are often limited [9,12,20,25,26,27,28,29].

Dosage and cycles of BV were also important for maintaining treatment efficacy while minimizing adverse events. The median number of BV retreatment cycles was 6.0 (IQR 2.0–20.0), with a mean of 7.6 cycles across all patients. These results are consistent with previous studies suggesting that six to eight cycles may strike the right balance between maximizing efficacy and reducing toxicity [21]. Notably, only 19% of patients required dose adjustments due to adverse events, mainly peripheral neuropathy, but these adjustments did not appear to compromise treatment outcomes. Previous studies have demonstrated that dose modifications tailored to patient toxicity profiles help to maintain both efficacy and tolerability [30].

The safety data from this study are consistent with the known safety profile of BV [1]. Peripheral neuropathy remains the most common adverse event, although only one patient needed a dose modification after BV retreatment. Moreover, the incidence of neuropathy grade 3–4 was relatively low, and treatment discontinuation due to AEs was infrequent. This suggests that with careful monitoring and dose adjustments, BV retreatment is feasible and well-tolerated, even in patients who have previously been exposed to BV.

Several potential sources of bias should be considered when interpreting the findings of this study. It is a retrospective study which encounters occasionally incomplete or inaccurate information, as well as variations based on heterogeneous practices derived from healthcare providers. However, these results provide evidence about the benefits of BV retreatment, which stimulates the development of prospective studies in larger cohorts to confirm these findings and to explore the potential benefits of BV retreatment in combination with other agents or as part of a maintenance therapy strategy.

## 5. Conclusions

The BELIEVE study demonstrates that BV retreatment can be an effective and well-tolerated option for patients with relapsed or refractory CD30-positive malignancies. The high response rates in cHL, sALCL, and CTCL patients, and the manageable safety profile, support the use of BV as a viable retreatment option. These findings contribute to the growing body of evidence supporting BV’s role in the early management of patients with CD30-positive lymphomas and provide a foundation for further research into optimizing treatment strategies for these challenging cases.

## Figures and Tables

**Table 1:** Demographic and baseline characteristics before BV retreatment.

		cHL (*n* = 16)	sALCL (*n* = 13)	CTCL (*n* = 14)	Total (*n* = 43)
Age at the time of visit (years)	Mean (SD)	36.2 (13.3)	51.3 (10.8)	52.9 (13.5)	46.2 (14.6)
	Min–Max	18.0–62.0	31.0–70.0	34.0–76.0	18.0–76.0
Gender	Male	9 (56.2%)	8 (61.5%)	8 (57.1%)	25 (58.1%)
	Female	7 (43.8%)	5 (38.5%)	6 (42.9%)	18 (41.9%)
Ethnicity	Caucasian	13 (86.7%)	12 (92.3%)	12 (85.7%)	37 (88.1%)
	Hispanic	2 (13.3%)	1 (7.7%)	1 (7.1%)	4 (9.5%)
	Other	0 (0%)	0 (0%)	1 (7.1%)	1 (2.4%)
	N missing	1	0	0	1
ECOG performance status at BV retreatment initiation	Grade 0	6 (50%)	7 (53.8%)	5 (41.7%)	18 (48.6%)
	Grade I	5 (41.7%)	5 (38.5%)	6 (50%)	16 (43.2%)
	Grade II	1 (8.3%)	0 (0%)	1 (8.3%)	2 (5.4%)
	Grade III	0 (0%)	1 (7.7%)	0 (0%)	1 (2.7%)
	Grade IV	0 (0%)	0 (0%)	0 (0%)	0 (0%)
	Grade V	0 (0%)	0 (0%)	0 (0%)	0 (0%)
	N missing	4	0	2	6
Clinical stage (Ann Arbor classification) at BV retreatment initiation	Stage I	1 (8.3%)	3 (23.1%)	-	4 (16%)
	Stage II	6 (50%)	2 (15.4%)	-	8 (32%)
	Stage III	2 (16.7%)	2 (15.4%)	-	4 (16%)
	Stage IVa	2 (16.7%)	2 (15.4%)	-	4 (16%)
	Stage IVb	1 (8.3%)	4 (30.8%)	-	5 (20%)
	N missing	4	0	-	18
Clinical stage (EORTC) at BV retreatment initiation	Stage IA	-	-	1 (9.1%)	1 (9.1%)
	Stage IB	-	-	1 (9.1%)	1 (9.1%)
	Stage IIA	-	-	0 (0%)	0 (0%)
	Stage IIB	-	-	3 (27.3%)	3 (27.3%)
	Stage III	-	-	0 (0%)	0 (0%)
	Stage IVA	-	-	2 (18.2%)	2 (18.2%)
	Stage IVB	-	-	4 (36.4%)	4 (36.4%)
	N missing	-	-	3	32

BV: Brentuximab vedotin; cHL: classical Hodgkin lymphoma; CTCL: cutaneous T-cell lymphoma; ECOG: Eastern Cooperative Oncology Group; EORTC: European Organisation for Research and Treatment of Cancer; sALCL: systemic anaplastic large cell lymphoma.

**Table 2:** Treatment patterns.

		cHL (*n* = 16)	sALCL (*n* = 13)	CTCL (*n* = 14)	Total (*n* = 43)
No. of treatments before BV retreatment	Mean (SD)	5.8 (3.1)	3.9 (2.1)	8.8 (6.9)	6.2 (4.8)
	Median	5.5	4.0	6.5	5.0
	(Min–Max)	(2.0–12.0)	(1.0–7.0)	(2.0–30.0)	(1.0–30.0)
Treatments between first BV and BV retreatment	No	3 (18.8%)	2 (15.4%)	2 (14.3%)	7 (16.3%)
	Yes	13 (81.2%)	11 (84.6%)	12 (85.7%)	36 (83.7%)
	Mean (SD)	1.5 (1.2)	1.5 (0.8)	1.6 (0.9)	1.6 (1.0)
	Median	1.0	1.0	1.0	1.0
	(Min–Max)	(1.0–5.0)	(1.0–3.0)	(1.0–3.0)	(1.0–5.0)
No. of treatments after BV retreatment	Mean (SD)	1.2 (2.4)	0.9 (0.9)	1.1 (1.8)	1.1 (1.8)
	Median	0.0	1.0	1.0	1.0
	(Min–Max)	(0.0–7.0)	(0.0–2.0)	(0.0–7.0)	(0.0–7.0)
No. of BV treatment cycles before BV retreatment	Mean (SD)	5.9 (4.4)	8.5 (4.6)	9.2 (4.0)	7.8 (4.5)
	Median	4.0	6.0	8.5	6.0
	(Min–Max)	(2.0;16.0)	(4.0;16.0)	(3.0;16.0)	(2.0;16.0)
No. of BV retreatment cycles	Mean (SD)	7.0 (5.4)	8.3 (5.5)	7.6 (3.7)	7.6 (4.8)
	Median	4.5	6.0	7.0	6.0
	(Min–Max)	(2.0–18.0)	(3.0–20.0)	(3.0–14.0)	(2.0–20.0)

BV: Brentuximab vedotin; cHL: classical Hodgkin lymphoma; CTCL: cutaneous T-cell lymphoma; sALCL: systemic anaplastic large cell lymphoma.

**Table 3:** Transplants before and after first BV treatment and retreatment.

		cHL (*n* = 16)	sALCL (*n* = 14)	CTCL (*n* = 13)	Total (*n* = 43)
No. of autologous transplants before first BV treatment	0	10 (62.5%)	11 (84.6%)	13 (92.9%)	34 (79.1%)
	1	6 (37.5%)	2 (15.4%)	1 (7.1%)	9 (20.9%)
	2	0 (0%)	0 (0%)	0 (0%)	0 (0%)
No. of autologous transplants after first BV treatment	0	11 (68.8%)	7 (53.8%)	14 (100%)	32 (74.4%)
	1	4 (25%)	6 (46.2%)	0 (0%)	10 (23.3%)
	2	1 (6.2%)	0 (0%)	0 (0%)	1 (2.3%)
No. of allogenic transplants before BV retreatment	0	11 (68.8%)	11 (84.6%)	13 (92.9%)	35 (81.4%)
	1	5 (31.2%)	1 (7.7%)	0 (0%)	6 (14%)
	2	0 (0%)	1 (7.7%)	1 (7.1%)	2 (4.7%)
No. of allogenic transplants after BV retreatment	0	12 (75%)	10 (76.9%)	12 (85.7%)	34 (79.1%)
	1	4 (25%)	3 (23.1%)	2 (14.3%)	9 (20.9%)
	2	0 (0%)	0 (0%)	0 (0%)	0 (0%)

BV: Brentuximab vedotin, cHL: classical Hodgkin lymphoma; CTCL: cutaneous T-cell lymphoma; sALCL: systemic anaplastic large cell lymphoma.

**Table 4:** Analysis for adverse events after BV retreatment.

		cHL	sALCL	CTCL	Total
AEs related to BV retreatment	Yes	7 (53.8%)	7 (53.8%)	4 (28.6%)	18 (45%)
	No	6 (46.2%)	6 (46.2%)	10 (71.4%)	22 (55%)
	N missing	3	0	0	3
Type of AEs per patient *	Peripheral Motor Neuropathy	2 (12.5%)	0 (0%)	0 (0%)	2 (4.7%)
	Peripheral Sensory Neuropathy	1 (6.2%)	5 (38.5%)	3 (21.4%)	9 (20.9%)
	Neutropenia	2 (12.5%)	2 (15.4%)	1 (7.1%)	5 (11.6%)
	Febrile Neutropenia	0 (0%)	0 (0%)	0 (0%)	0 (0%)
	Renal dysfunction	0 (0%)	0 (0%)	0 (0%)	0 (0%)
	Liver dysfunction	0 (0%)	0 (0%)	0 (0%)	0 (0%)
	Pulmonary disorder	0 (0%)	1 (7.7%)	0 (0%)	1 (2.3%)
	Others with clinical relevance	4 (25%)	5 (38.5%)	1 (7.1%)	10 (23.3%)
Severity of the AE per patient *	Severe	2 (12.5%)	3 (23.1%)	3 (21.4%)	8 (18.6%)
	Non-Severe	6 (37.5%)	7 (53.8%)	2 (14.3%)	15 (34.9%)
	N valid	8	10	5	23

* Percentage respect to the overall patients for each cohort. AE: adverse event; cHL: classical Hodgkin lymphoma; CTCL: cutaneous T-cell lymphoma; sALCL: systemic anaplastic large cell lymphoma.

## Data Availability

The raw data supporting the conclusions of this article will be made available by the authors on request.

## Supplementary Materials

The following supporting information can be downloaded at https://www.mdpi.com/article/10.3390/cancers17071137/s1, Figure S1: Responses and duration of the treatment and retreatment with BV periods for cHL, sALCL and CTCL patients.
