# Supplementary material for: Retreatment with Brentuximab Vedotin in Patients with Relapsed/Refractory CD30+ Malignancies: A Retrospective Medical Chart Review Study in Spain-The BELIEVE Study
Source: Cancers (Basel). 2025 Mar 28;17(7):1137. doi: 10.3390/cancers17071137 (PMC11987772; doi:10.3390/cancers17071137)
Supplement: Supplementary file 1 [file cancers-17-01137-s001.zip › cancers-3512747-supplementary.pdf]

Supplementary material

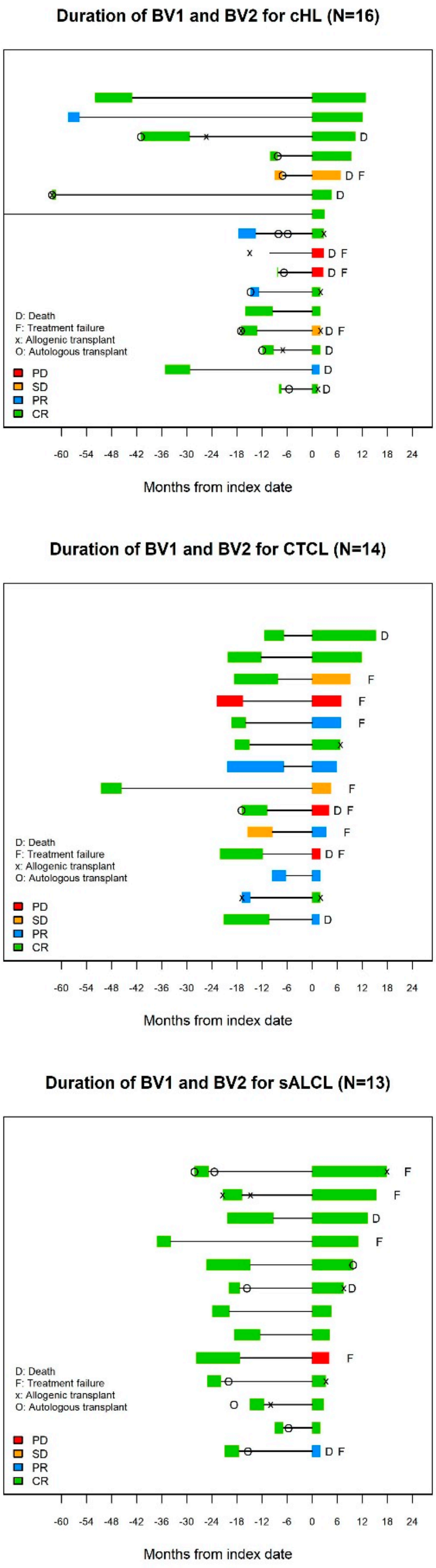

Figure S1. Responses and durations of the treatments and retreatments, with BV periods, for cHL, sALCL and CTCL patients.
